# Heart Point in Pneumothorax: A Case Report and Comparison to Mimicking Signs

**DOI:** 10.1155/crcc/8134006

**Published:** 2025-11-19

**Authors:** Veerle Verhees, Laura G. C. de Kok

**Affiliations:** ^1^ Department of Intensive Care, Erasmus Medisch Centrum, Rotterdam, the Netherlands

**Keywords:** echocardiography, heart point, pneumothorax, point-of-care ultrasound

## Abstract

**Background:**

Lung ultrasound is increasingly used in critical care medicine to identify pneumothorax, usually by the absence of lung sliding and identification of the lung point. Heart point is a less recognized but specific sign in cardiac ultrasound indicating pneumothorax.

**Case Presentation:**

A 20‐year‐old Caucasian male presented with a pneumothorax after a traumatic resuscitation to the emergency room upon bilateral chest decompression being initiated. Upon presentation to the intensive care unit, there were no clinical signs of pulmonary compromise on mechanical ventilation. Performance of transthoracic echography at the ICU identified heart point, and the subsequent diagnosis of pneumothorax was made. A thoracic drain was inserted and resulted in the re‐expansion of the lung. The chest tube was removed after 2 days.

**Conclusion:**

The utility of point‐of‐care ultrasound in the diagnosis of pneumothorax is aided by awareness of heart point. Recognition is imperative to ensure prompt therapeutic measures and ensure improved patient outcomes. Knowledge of mimicking appearances such as pseudo heart point and air gap sign is essential to assure diagnostic accuracy.

## 1. Introduction

Pneumothorax is a critical condition that can present significant diagnostic challenges, especially when conventional imaging modalities are either unavailable or delayed. Early and accurate identification is crucial for ensuring prompt and appropriate treatment, particularly in critically ill patients, such as those on mechanical ventilation. In these scenarios, a delay in diagnosis can result in severe complications, including respiratory failure, cardiovascular collapse, or even death [1]. Given these risks, it is essential to implement rapid diagnostic techniques that facilitate timely interventions.

Point‐of‐care ultrasound (POCUS) has gained recognition as an effective diagnostic modality for pneumothorax in the intensive care unit (ICU) setting. This technique is noninvasive, portable, and can be performed rapidly at the patient’s bedside, eliminating the need for transport and facilitating immediate decision‐making. Lung ultrasound has been shown to be highly accurate in diagnosing pneumothorax, with several sonographic signs demonstrating strong sensitivity and specificity [2]. These signs include the absence of lung sliding (the movement of the pleura against the chest wall during respiration) and B‐lines [3], and the “lung point” (which marks the location where the pleura begins to move after a portion of the lung is no longer in contact with the chest wall) [4].

In addition to these well‐established signs, the “heart point” sign has emerged as a valuable diagnostic indicator for pneumothorax [5]. The heart point sign refers to the transient loss of the cardiac image during the cardiac cycle, caused by the displacement of air in the pleural space due to cardiac contraction. This phenomenon occurs when the heart, which moves closer to the chest wall during diastole, is temporarily obscured by air in the pleural space during systole, when the heart moves away from the chest wall. The heart point is particularly useful for detecting ventrally located pneumothoraces, which are often missed on traditional anteroposterior chest X‐rays, especially in patients who are supine [6]. Recognizing this phenomenon is crucial for the timely identification of ventral pneumothorax, ensuring prompt therapeutic intervention and preventing further complications.

This case report presents a patient with traumatic brain injury who developed an unexpected pneumothorax, diagnosed using the heart point sign after initial resuscitation and bilateral chest decompression. By illustrating the diagnostic utility of the heart point sign in this case, we emphasize the role of bedside ultrasound as a valuable diagnostic tool, especially in settings where immediate access to conventional imaging techniques may be limited. Furthermore, we highlight the importance of differentiating the heart point from other sonographic phenomena that may mimic similar findings, ensuring accurate diagnosis and appropriate management.

## 2. Case Presentation

A 20‐year‐old male with no significant medical history sustained blunt trauma as a pedestrian struck by a vehicle in a high‐velocity collision. Upon arrival of the Helicopter Emergency Medical Services (HEMS), the patient was in traumatic cardiac arrest. Advanced trauma life support was promptly initiated, including endotracheal intubation and resuscitation with large volumes of intravenous fluids. In accordance with the ERC guidelines on traumatic cardiac arrest [7], the HEMS doctor performed bilateral thoracostomy in order to relieve possible pneumothorax as a very common reversible cause. The presence of subcutaneous emphysema underlined this likely cause. Following bilateral chest decompression via thoracostomy, the patient regained spontaneous circulation at the scene.

**Figure 1:**
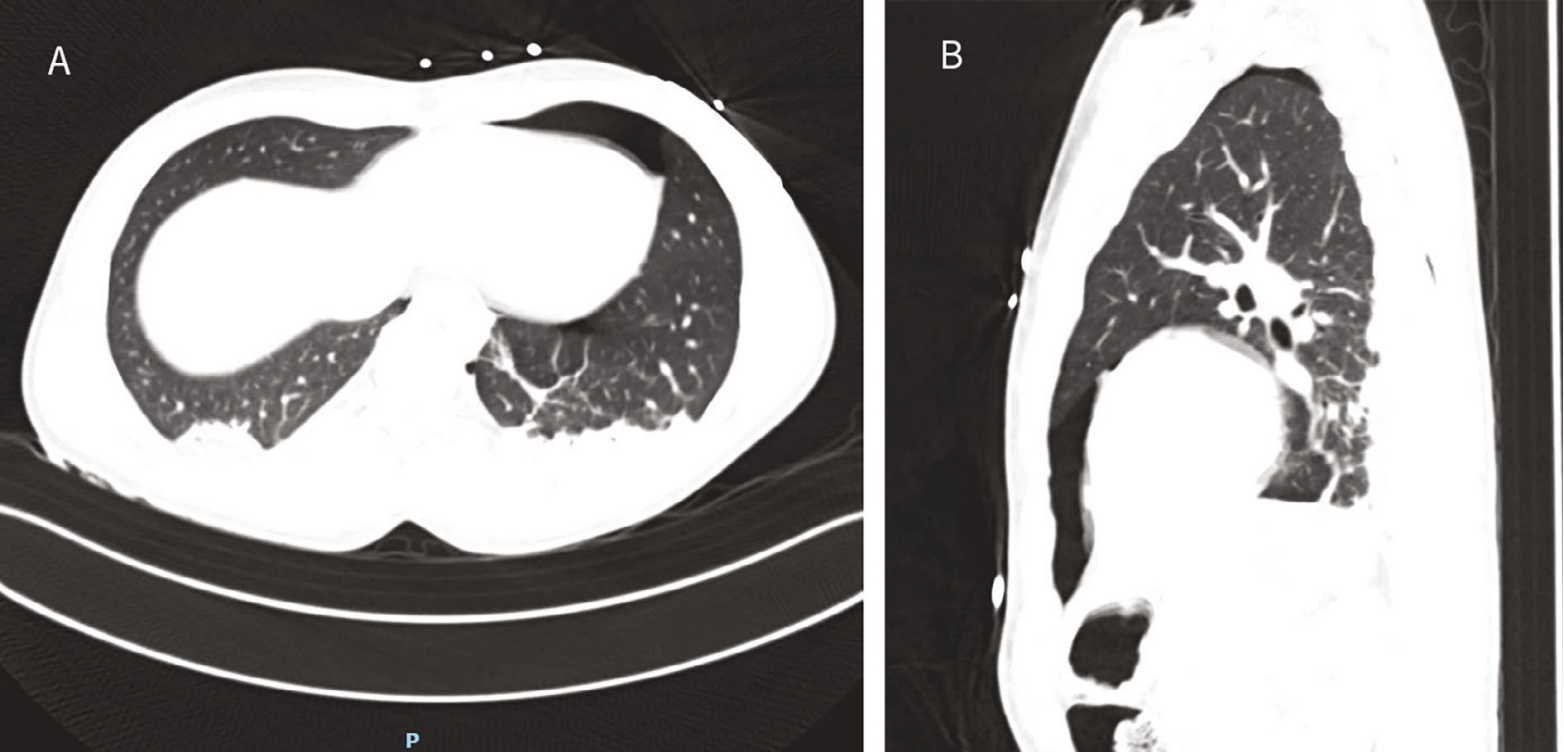
Thoracic CT scan made in the emergency department, showing a small ventral left‐sided pneumothorax. (A) Axial view. (B) Sagittal view.

Upon arrival at the emergency department, the patient was stabilized. A thoracic computed tomography (CT) scan revealed a small left‐sided ventral pneumothorax, which was managed conservatively with observation due to its limited size (Figure 1), the low ventilator settings, and the ability to monitor the patient closely in the ICU. The thoracostomy wounds were closed with simple sutures, and subsequent imaging confirmed no immediate life‐threatening concerns. Other injuries identified by CT scan were bilateral ground glass opacities, which indicated possible lung contusion, a fracture of the transverse process of the L1 vertebra, and a fracture of the facet of the C6 vertebra. The patient was then admitted to the ICU for further monitoring and management.

At the time of ICU admission, the patient was sedated with midazolam (0.15 mg/kg/h) and sufentanil (0.7 mcg/kg/h). An intracranial pressure (ICP) monitor was inserted to monitor for secondary brain injury, and the ICP readings remained low. Mechanical ventilation was uncomplicated and well tolerated with low settings (tidal volumes set at 6 mL/kg, plateau pressure 15 cm H_2_O, driving pressure 10 cm H_2_O), ensuring adequate oxygenation and ventilation. Hemodynamically, the patient′s heart rate was 60 beats per min, and blood pressure was 110/50 mmHg, supported by noradrenaline infusion at a rate of 0.15 mcg/kg/min.

The electrocardiogram performed upon ICU admission revealed an increase in premature atrial complexes and variable atrial ectopy. Consequently, the intensivist decided to perform a comprehensive transthoracic echocardiographic examination using a 4–2‐MHz sector array transducer (Philips S4‐2) to rule out traumatic cardiac complications. However, adequate visualization of the heart was not possible due to transient disappearance of the heart during systole in both the parasternal short axis (Figure 2 and Video S1) and parasternal long axis views (Video S2). This phenomenon raised suspicion for the presence of a pneumothorax. The apical view could not be obtained. In contrast, the heart was clearly visible throughout the cardiac cycle in the subxiphoid view (Video S3).

Figure 2Still of Video S1. Parasternal short axis (PSAX) view of the heart. (a) Visualization of the heart during diastole. (b) Loss of visualization of the heart during systole. Furthermore, A‐lines can be seen during systole, indicating air in the thoracic cavity. No pericardium can be seen during systole, indicating pneumothorax rather than pneumopericardium.

**Figure:**
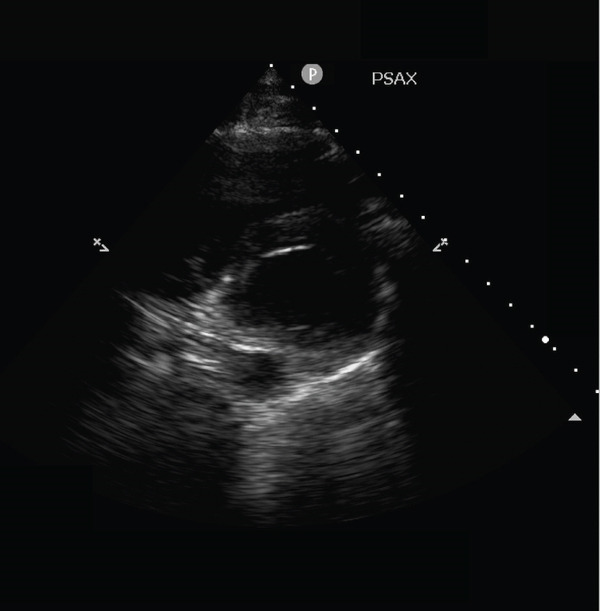
(a)

**Figure:**
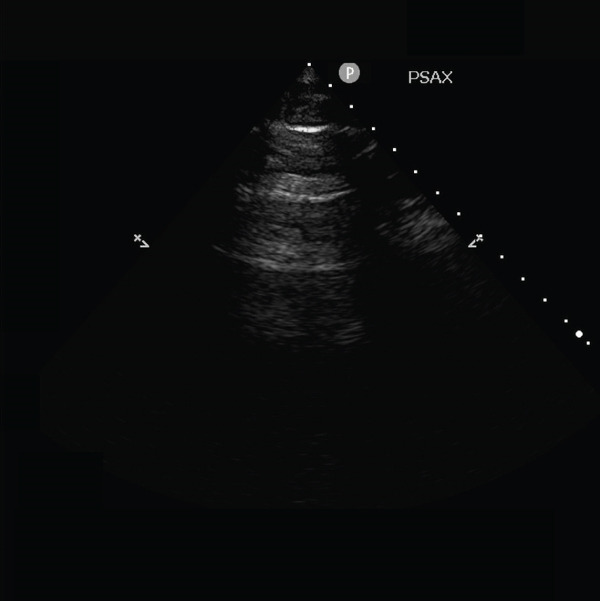
(b)

To further investigate, a lung ultrasound was performed using a 12–4‐MHz linear transducer (Philips L12‐4). Right‐sided lung sliding was observed, whereas it was absent on the left side. The lung point was identified in the midclavicular plane at the fourth intercostal space (Video S4) and could be traced cranially. When comparing the anatomical location of the lung point on ultrasound to prior imaging of the thorax on CT scan, this was indicative of an increase in the left‐sided ventral pneumothorax.

The diagnosis was further corroborated by chest radiography, which demonstrated a deep sulcus sign on the left and a small, clinically insignificant pneumothorax on the right. A left thoracostomy tube was placed at the site of previous chest decompression. Follow‐up chest radiography showed improved lung expansion and a reduction in the size of the pneumothorax. Once the lung had fully re‐expanded, the thoracostomy tube was removed after 2 days, with no pulmonary sequelae.

The patient continued to recover from his traumatic brain injury and was successfully discharged from the ICU after 2 weeks.

## 3. Discussion

In this case, worsening of pneumothorax severity was incidentally diagnosed during POCUS of the heart through the recognition of the “heart point sign.” Awareness and accurate identification of this sonographic finding are crucial for facilitating prompt therapeutic intervention. Given the widespread integration of POCUS in critical care settings, including the ICU and emergency department, clinicians must be proficient in recognizing ultrasound features indicative of pneumothorax to prevent diagnostic delays or unnecessary invasive procedures.

Evidence supports the use of lung ultrasound as a reliable, sensitive, and specific tool for the diagnosis of pneumothorax, based on the recognition of absent lung sliding, lung pulse, and B‐lines and the presence of lung point [8]. A Cochrane systematic review confirmed that lung ultrasound outperforms chest radiography in both sensitivity and specificity for traumatic pneumothorax detection, especially in critically ill patients [9]. Similarly, a recent review has emphasized that the method is not only validated but also practical and reproducible in routine clinical practice [10]. These findings underscore that lung ultrasound is well established as a frontline diagnostic modality, and this case report highlights additional, less conventional signs that can further aid diagnosis in challenging clinical scenarios.

Previous case reports have described sonographic indicators of pneumothorax beyond the conventional absence of lung sliding and presence of a lung point, which signifies the separation of the visceral and parietal pleura. The heart point sign was first documented by Stone et al. in a parasternal view during an emergency department evaluation, where it provided an unexpected yet critical clue to the diagnosis [5]. Subsequent reports by Cheong et al. expanded upon this finding, demonstrating the heart point sign in both parasternal and apical views using M‐mode while assessing tricuspid annular plane systolic excursion [11, 12].

A key challenge in recognizing the heart point sign lies in distinguishing it from other sonographic artifacts, such as the pseudo‐heart point, which arise due to respiratory rather than cardiac cycles [13]. The pseudo‐heart point represents a physiological phenomenon in which the heart disappears from view during inspiration as the lung expands and displaces cardiac structures. In contrast, the heart point sign is characterized by cardiac motion‐dependent changes in heart visibility, a feature integral to diagnosing pneumothorax.

The underlying principle of cardiac visibility alteration due to air displacement was initially described in 1983 in cases of pneumomediastinum and pneumopericardium, a phenomenon referred to as the “air gap sign” [14]. The air gap sign closely resembles the heart point, as both result from air obscuring cardiac structures during systole. However, in pneumomediastinum, the air accumulates within the mediastinal space rather than the pleural cavity, distinguishing it from pneumothorax. While pneumomediastinum and pneumopericardium are often self‐limiting conditions, pneumothorax presents a more immediate clinical risk, necessitating urgent decompression in severe cases.

The differentiation between the heart point sign in pneumothorax and the air gap sign in pneumopericardium can be achieved through careful sonographic evaluation of the pericardium. In pneumopericardium, the hyperechoic pericardial layer remains visible throughout the cardiac cycle, whereas in pneumothorax, it disappears during systole. Additionally, pneumopericardium may generate erratic comet‐tail artifacts originating from the pericardium, likely due to the interaction between an anechoic pericardial fluid layer and an air‐fluid interface [15].

Zachariah et al. further elaborated on the distinction between pneumomediastinum and pneumopericardium using cardiac ultrasound [16]. It was proposed that in pneumopericardium, all cardiac ultrasound windows, including the subxiphoid view, may be obscured by air during systole, resulting in the air gap sign. Conversely, in pneumomediastinum, the heart maintains contact with the diaphragm and remains visible in the subxiphoid view throughout the cardiac cycle, despite exhibiting the air gap sign in other windows. This similarity complicates differentiation between the heart point sign in pneumothorax and the air gap sign in pneumomediastinum, necessitating comprehensive ultrasound assessment. Differentiating the still lung point (which does not move with respiration) indicating pneumomediastinum from the true lung point indicating pneumothorax can help to further differentiate [17].

In the present case, the heart point sign was observed exclusively in parasternal views, while the heart remained visible in the subxiphoid window throughout the cardiac cycle, closely resembling pneumomediastinum. This highlights the critical importance of confirming pneumothorax by identifying the lung point, as large pneumomediastinum can also be associated with absent lung sliding [18].

In conclusion, the heart point sign represents a valuable sonographic indicator of pneumothorax that critical care physicians should recognize to facilitate timely diagnosis and intervention. Differentiating the heart point from similar ultrasound artifacts, such as the pseudo‐heart point and the air gap sign seen in pneumomediastinum and pneumopericardium, is essential to avoid misdiagnosis. While lung ultrasound has been validated as a reliable and accurate diagnostic tool for pneumothorax, recognition of additional signs such as the heart point further enhances diagnostic confidence and reinforces the role of ultrasound as a cornerstone in critical care imaging. When the heart point is identified, clinicians should have a high index of suspicion for pneumothorax, and appropriate management—including chest tube placement—should be considered in conjunction with clinical findings and confirmatory ultrasound features.

## Consent

Written informed consent was obtained from both parents of the patient. The case report was written following the CARE guidelines [19].

## Conflicts of Interest

The authors declare no conflicts of interest.

## Funding

No funding was received for this manuscript.

## Supporting Information

Additional supporting information can be found online in the Supporting Information section.

## Supporting information


**Supporting Information 1** Video S1: Heart point sign in the parasternal short axis view of the heart. The heart and pericardium vanish from view during systole and become visible during diastole. A‐lines can be seen when the heart is not visible.

**Supporting Information 2** Video S2: Heart point sign in the parasternal long axis view of the heart.

**Supporting Information 3** Video S3: Subxiphoid view of the heart. The heart remains visible during the full heart cycle.

**Supporting Information 4** Video S4: Lung point confirming the diagnosis of pneumothorax.

## Data Availability

The data that support the findings of this study are available from the corresponding author upon reasonable request.
